# Supplementary material for: From sequence to enzyme mechanism using multi-label machine learning
Source: BMC Bioinformatics. 2014 May 19;15:150. doi: 10.1186/1471-2105-15-150 (PMC4229970; doi:10.1186/1471-2105-15-150)
Supplement: Additional file 2 — Java code of ml2db. Additional file ml2db_code.tar.gz contains the Java source code to run the multi-label machine learning experiments and save the results to database. The code’s Javadoc is included. [file 1471-2105-15-150-S2.zip › additional file 2/ml2db/ml2db/doc/mulan/classifier/neural/BPMLL.html]

BPMLL


JavaScript is disabled on your browser.


- Overview
- Package
- Class
- Use
- Tree
- Deprecated
- Index
- Help

- Prev Class
- Next Class

- Frames
- No Frames

- All Classes

- Summary:
- Nested |
- Field |
- Constr |
- Method

- Detail:
- Field |
- Constr |
- Method


mulan.classifier.neural

## Class BPMLL

- java.lang.Object
- - mulan.classifier.MultiLabelLearnerBase
  - - mulan.classifier.neural.BPMLL

- All Implemented Interfaces:
  :   java.io.Serializable, MultiLabelLearner, weka.core.TechnicalInformationHandler

  ---

    

  ```
  public class BPMLL
  extends MultiLabelLearnerBase
  ```

  The implementation of Back-Propagation Multi-Label Learning (BPMLL) learner. The learned model is stored in {@link NeuralNet} neural network. The models of the learner built by {@link BPMLLAlgorithm} from given training data set.


  BibTeX:

  ```
   @article{Zhang2006,
      author = {Zhang, M.L., Zhou, Z.H.},
      journal = {IEEE Transactions on Knowledge and Data Engineering},
      pages = {1338-1351},
      title = {Multi-label neural networks with applications to functional genomics and text categorization},
      volume = {18},
      year = {2006}
   }
  ```

  Version:
  :   2012.02.27

  Author:
  :   Jozef Vilcek

  See Also:
  :   `BPMLLAlgorithm`,
      Serialized Form

- - ### Constructor Summary

    Constructors

    Constructor and Description

    `BPMLL()`

    Creates a new instance of `BPMLL` learner.

    `BPMLL(long randomnessSeed)`

    Creates a new instance of `BPMLL` learner.
  - ### Method Summary

    Methods

    | Modifier and Type | Method and Description |
    | --- | --- |
    | `int[]` | `getHiddenLayers()` Gets an array defining topology of hidden layer of the underlying neural model. |
    | `double` | `getLearningRate()` Gets the learning rate. |
    | `boolean` | `getNormalizeAttributes()` Gets a value if normalization of nominal attributes should take place. |
    | `weka.core.TechnicalInformation` | `getTechnicalInformation()` Returns an instance of a TechnicalInformation object, containing detailed information about the technical background of this class, e.g., paper reference or book this class is based on. |
    | `int` | `getTrainingEpochs()` Gets number of training epochs. |
    | `double` | `getWeightsDecayRegularization()` Gets a value of the regularization cost term for weights decay. |
    | `java.lang.String` | `globalInfo()` Returns a string describing the multi-label learner. |
    | `MultiLabelOutput` | `makePredictionInternal(weka.core.Instance instance)` Learner specific implementation for predicting on specified data based on trained model. |
    | `void` | `setHiddenLayers(int[] hiddenLayers)` Sets the topology of hidden layers for neural network. |
    | `void` | `setLearningRate(double learningRate)` Sets the learning rate. |
    | `void` | `setNormalizeAttributes(boolean normalize)` Sets whether attributes of instances data (except label attributes) should be normalized prior to building the learner. |
    | `void` | `setTrainingEpochs(int epochs)` Sets the number of training epochs. |
    | `void` | `setWeightsDecayRegularization(double weightsDecayCost)` Sets the regularization cost term for weights decay. |

    - ### Methods inherited from class mulan.classifier.MultiLabelLearnerBase

      `build, getDebug, isUpdatable, makeCopy, makePrediction, setDebug`
    - ### Methods inherited from class java.lang.Object

      `equals, getClass, hashCode, notify, notifyAll, toString, wait, wait, wait`

- - ### Constructor Detail


    - #### BPMLL

      ```
      public BPMLL()
      ```

      Creates a new instance of `BPMLL` learner.


    - #### BPMLL

      ```
      public BPMLL(long randomnessSeed)
      ```

      Creates a new instance of `BPMLL` learner.

      Parameters:
      :   `randomnessSeed` - the seed value for pseudo-random generator
  - ### Method Detail


    - #### setHiddenLayers

      ```
      public void setHiddenLayers(int[] hiddenLayers)
      ```

      Sets the topology of hidden layers for neural network.
      The length of passed array defines number of hidden layers.
      The value at particular index of array defines number of neurons in that layer.
      If `null` is specified, no hidden layers will be created.

      Parameters:
      :   `hiddenLayers` -

      Throws:
      :   `java.lang.IllegalArgumentException` - if any value in the array is less or equal to zero


    - #### getHiddenLayers

      ```
      public int[] getHiddenLayers()
      ```

      Gets an array defining topology of hidden layer of the underlying neural model.

      Returns:
      :   The method returns a copy of the array.


    - #### setLearningRate

      ```
      public void setLearningRate(double learningRate)
      ```

      Sets the learning rate. Must be greater than 0 and no more than 1.  
      Default value is 0.05.

      Parameters:
      :   `learningRate` - the learning rate

      Throws:
      :   `java.lang.IllegalArgumentException` - if passed value is invalid


    - #### getLearningRate

      ```
      public double getLearningRate()
      ```

      Gets the learning rate. The default value is 0.05.

      Returns:
      :   learning rate


    - #### setWeightsDecayRegularization

      ```
      public void setWeightsDecayRegularization(double weightsDecayCost)
      ```

      Sets the regularization cost term for weights decay.
      Must be greater than 0 and no more than 1.  
      Default value is 0.00001.

      Parameters:
      :   `weightsDecayCost` - the weights decay cost term

      Throws:
      :   `java.lang.IllegalArgumentException` - if passed value is invalid


    - #### getWeightsDecayRegularization

      ```
      public double getWeightsDecayRegularization()
      ```

      Gets a value of the regularization cost term for weights decay.

      Returns:
      :   regularization cost


    - #### setTrainingEpochs

      ```
      public void setTrainingEpochs(int epochs)
      ```

      Sets the number of training epochs. Must be greater than 0.  
      Default value is 100.

      Parameters:
      :   `epochs` - the number of training epochs

      Throws:
      :   `java.lang.IllegalArgumentException` - if passed value is invalid


    - #### getTrainingEpochs

      ```
      public int getTrainingEpochs()
      ```

      Gets number of training epochs.
      Default value is 100.

      Returns:
      :   training epochs


    - #### setNormalizeAttributes

      ```
      public void setNormalizeAttributes(boolean normalize)
      ```

      Sets whether attributes of instances data (except label attributes) should
      be normalized prior to building the learner. Normalization is performed
      on numeric attributes to the range {-1,1}).  
      When making prediction, attributes of passed input instance are also
      normalized prior to making prediction.  
      Default is true (normalization of attributes takes place).

      Parameters:
      :   `normalize` - flag if normalization of attributes should be used

      Throws:
      :   `java.lang.IllegalArgumentException` - if passed value is invalid


    - #### getNormalizeAttributes

      ```
      public boolean getNormalizeAttributes()
      ```

      Gets a value if normalization of nominal attributes should take place.
      Default value is true.

      Returns:
      :   a value if normalization of nominal attributes should take place


    - #### globalInfo

      ```
      public java.lang.String globalInfo()
      ```

      **Description copied from class: `MultiLabelLearnerBase`**

      Returns a string describing the multi-label learner.

      **Specified by:**
      :   `globalInfo` in class `MultiLabelLearnerBase`

      Returns:
      :   a description suitable for displaying in a future gui


    - #### getTechnicalInformation

      ```
      public weka.core.TechnicalInformation getTechnicalInformation()
      ```

      **Description copied from class: `MultiLabelLearnerBase`**

      Returns an instance of a TechnicalInformation object, containing detailed
      information about the technical background of this class, e.g., paper
      reference or book this class is based on.

      **Specified by:**
      :   `getTechnicalInformation` in interface `weka.core.TechnicalInformationHandler`

      **Specified by:**
      :   `getTechnicalInformation` in class `MultiLabelLearnerBase`

      Returns:
      :   the technical information about this class


    - #### makePredictionInternal

      ```
      public MultiLabelOutput makePredictionInternal(weka.core.Instance instance)
                                              throws InvalidDataException
      ```

      **Description copied from class: `MultiLabelLearnerBase`**

      Learner specific implementation for predicting on specified data based on trained model.
      This method is called from `MultiLabelLearnerBase.makePrediction(weka.core.Instance)` which guards for model
      initialization and apply common handling/behavior.

      Parameters:
      :   `instance` - the data instance to predict on

      Returns:
      :   the output of the learner for the given instance

      Throws:
      :   `InvalidDataException` - if specified instance data is invalid and can not be processed by the learner


- Overview
- Package
- Class
- Use
- Tree
- Deprecated
- Index
- Help

- Prev Class
- Next Class

- Frames
- No Frames

- All Classes

- Summary:
- Nested |
- Field |
- Constr |
- Method

- Detail:
- Field |
- Constr |
- Method
